# The association of illness perception and related factors with treatment adherence among chronic hemodialysis patients with cardio-renal syndrome in Yemen

**DOI:** 10.3389/fcvm.2025.1432648

**Published:** 2025-04-30

**Authors:** Adel Omar Laradhi, Yan Shan, Abdulaziz Mansoor Al Raimi, Nahed Ahmed Hussien, Eman Ragab, Mikiyas Amare Getu, Galal Al-Bani, Mohamed Elsayed Allawy

**Affiliations:** ^1^ Department of Medical Surgical Nursing, College of Nursing, University of Hail, Hail, Saudi Arabia; ^2^ The Third Affiliated Hospital of Zhengzhou University, Zhengzhou, Henan, China; ^3^ Seiyun Community College, Hadhramout, Yemen; ^4^ Faculty of Nursing, Suez Canal University, Ismailia, Egypt; ^5^ School of Nursing and Health, Woldia University, Woldia, Ethiopia; ^6^ Department of Nursing Sciences, College of Applied Medical Sciences, Prince Sattam bin Abdulaziz University, Wadi Alddawasir, Saudi Arabia

**Keywords:** cardio-renal syndrome, hemodialysis, illness perception, treatment adherence, Yemen

## Abstract

**Background:**

Patients’ illness perceptions are cognitive representations or beliefs structured around their condition. These perceptions have been associated with several important outcomes, including functional recovery and treatment adherence.

**Objective:**

The aim of this study was to investigate the association between illness perception and related factors with treatment adherence among hemodialysis patients with cardio-renal syndrome in Yemen.

**Methods:**

This cross-sectional study was conducted at two dialysis centers in Hadhramout Governorate, Yemen, from February to May 2021. Three self-administered questionnaires were used to collect the data. Data analysis was performed using SPSS version 23.0 with a significance level set at *p* < 0.05.

**Results:**

In total, 100 patients answered all questions with a total response rate of 100%. The mean age ± standard deviation of participants was 53.46 ± 14.24 years. Most patients (87%) had a low level of treatment adherence, particularly in medication and diet restriction adherence. Furthermore, most patients (90%) had a moderate-to-high level of perceived illness threat. The findings revealed no significant correlation between overall illness perception and overall treatment adherence (*r* = 0.003, *p* = 0.975). However, the perceived consequences (*r* = −0.210, *p* < 0.05), and perceived timeline subscales (*r* = −0.276, *p* < 0.01) showed a negative correlation with total treatment adherence. Additional findings revealed a significant positive link between adherence and cardiac disorders (*β* = 4.292, *p* = 0.009), as well as a strong correlation between adherence and income level (*β* = 11.132, *p* < 0.001).

**Conclusions:**

Our research found that most patients with cardio-renal syndrome had poor treatment adherence and had a moderate-to-high level of perceived illness threat. The results of the study showed that perceived consequences and perceived timeline subscales of illness perceptions had a negative correlation with overall treatment adherence, and the results revealed a significant positive link between adherence and cardiac disorders, as well as a strong correlation between adherence and income level. The findings suggest that nurses and clinicians should assess the illness perceptions specific to patients with cardio-renal syndrome disease when developing multidisciplinary interventions to help patients cope with and manage different aspects of their condition.

## 1 Introduction

Cardio-renal syndrome (CRS) is acute or chronic heart or kidney malfunction that can cause immediate or chronic dysfunction in the other organ. Both heart and kidney function can be impaired by an acute or chronic systemic disorder (1), causing a combination of hemodynamic mechanisms, oxidative stress, neuro-hormonal activation, hypothalamus-pituitary stress, inflammation, anemia, and iatrogenic causes (2). Cardiovascular disease (CVD) is responsible for more than half of all fatalities in patients with chronic kidney disease (CKD) and is the main cause of death. Furthermore, chronic heart failure (HF) affects up to 30% of CKD patients (3, 4). Moreover, CKD affects 30%–50% of all patients with HF and is a potent risk factor for cardiovascular death and HF hospitalization (5). Therefore, both cardiac and renal diseases should be managed effectively to prevent or reduce the development or worsening of the adverse effects.

The healthcare system in Yemen has been severely compromised following 6 years of prolonged conflict. Approximately half of the nation's health facilities are currently inoperable, and the majority of those that are operational have severely restricted capabilities (6). The majority of the population is unable to access healthcare as a result of the devastation of health facilities in their respective regions or a lack of financial resources (7). The aforementioned variables often exacerbate the distress experienced by CRS patients, leading them to perceive minimal healthcare quality as adequate health services.

Successful renal replacement therapy (RRT) for patients with end-stage renal disease (ESRD) depends on proper patient self-care through adherence to significant aspects of the therapy, such as session attendance, fluid restriction, dietary modalities, and adherence to complex and established medical guidelines (8). Adherence to a treatment plan is acknowledged as a global health issue, with special significance in the care of cardiovascular patients (9). Patient adherence to the prescribed course of treatment is a crucial sign that chronic diseases are well-controlled (10) and non-adherence in ESRD is strongly correlated with poorer quality of life, increased mortality, and increased morbidity, including impaired physical abilities, depression, acute pulmonary edema, and congestive heart failure (11, 12). Early patient identification of those at risk for adverse outcomes can benefit from the knowledge of non-adherence risk and resiliency variables (13). Treatment adherence should be improved in this population to prevent and reduce the development of CRS and/or adverse effects. The early identification of poor treatment adherence and its associated factors is critically important.

Illness perceptions are a factor potentially affecting treatment adherence. The common-sense model (CSM) created by Leventhal et al. suggest a relationship between illness perceptions and treatment adherence (14). The CSM model proposes that individuals construct mental representations of a specific condition, which determines the coping behaviors and procedures used to manage the condition. In the CSM, illness perception denotes how a person conceptualizes and mentally frames living with the disease (14, 15). Although perceptions of illness have been the core of previous research (16–22), no studies have examined the relationship in patients with CRS. Culture-specific information contributes to the perception or construction of perception (23). However, no studies have examined the status of illness perceptions and treatment adherence among patients with CRS in Yemen. Illness perception and treatment adherence in this population may be influenced by receiving hemodialysis treatment. Therefore, the purpose of this study was to assess the levels of illness perception and treatment adherence and to examine the relationships between illness perception and related factors with treatment adherence among hemodialysis patients with CRS.

## 2 Materials and methods

### 2.1 Design and setting

A descriptive cross-sectional design was used to accomplish the study's aim. Patients were enrolled during regular visits to two dialysis centers in Hadhramout City, Yemen, from February to May 2021.

### 2.2 Procedure and participants

The criteria for inclusion were as follows: Yemeni patients diagnosed with any type of cardio-renal syndrome for at least 6 months and confirmed by the investigators, aged 18 years or older, able to read and comprehend the Arabic language, and underwent hemodialysis treatment three times per week for at least 3 months prior to study admission based on medical chart review. CRS mutation carriers and patients who had a psychiatric or cognitive disease were excluded. Patients were enrolled in the study using a convenience sampling technique. The sample size was calculated using a single proportion formula at a 95% confidence level (*Z*-score of 1.96) with a margin of error (E) of 0.1 and an estimated population proportion (p) of 0.5 (24). The initial calculated sample size was 97 participants. To account for a potential 5% non-response rate, the final sample size was adjusted to 100. Ultimately, 100 patients with CRS who attended dialysis centers during the study period and agreed to participate were included in the study.

### 2.3 Materials

The data were collected using three self-administered questionnaires:

#### 2.3.1 The Demographic and Clinical Data Questionnaire

The researchers developed the Demographic and Clinical Data Questionnaire (DCDQ). This questionnaire has two sections: the first section has eight questions to assess participants’ demographic characteristics, including age, sex, marital status, educational level, income, occupation, no. of family members, and daily diet prepared. The second section has four questions to assess the patients’ clinical data.

#### 2.3.2 The Brief Illness Perception Questionnaire

The Brief Illness Perception Questionnaire (Brief IPQ-Ar) was used to assess the patients’ illness perception among patients with CRS after obtaining formal permission from the author. Responses are assessed using a scale from 0 to 10. For items 1, 2, 5, 6, and 8, a 0 score indicates a positive illness perception and a score of 10 indicates a negative illness perception. For items 3, 4, and 7, a score of 0 indicates a negative illness perception, and 10 indicates a positive illness perception. Assessment of the causal representation was conducted through an open-ended response item, which asked the patients to list the three most important causal factors in their illness (item 9). The total illness perception scores were computed by summing up the scores for items 1–8 after reverse scoring items 3, 4, and 7. The range of possible (or potential) total scores is 0–80. A higher score reflects worse illness perception. For further interpretation of the Brief IPQ, the scores were divided into three groups: 0–27 was considered a low level of perceived illness threat, 28–55 was considered a moderate level of perceived illness threat, and 56–80 was considered a high level of perceived illness threat (25). The responses to item 9 were analyzed by categorizing the causes and utilizing descriptive statistics. We chose the Brief IPQ for its rapid assessment of illness perceptions, which is particularly beneficial for seriously ill patients (26). It has been validated in large-scale studies and is suitable for Arabic-speaking cardiac disease patients. The questionnaire was adopted from a previous Arabic study (27) and the Cronbach's alpha for the Brief IPQ-Ar was 0.79, which can be regarded as a good value.

#### 2.3.3 The Treatment Adherence Questionnaire

The Treatment Adherence Questionnaire (TAQ) was used to evaluate treatment adherence in patients with CRS dependence on dialysis. This instrument includes dialysis adherence, medication adherence, fluid restriction adherence, and diet restriction adherence. The questionnaire contains 15 items, and the response options range from 1 to 4 on a 4-point Likert scale (1 = never, 2 = sometimes, 3 = most of the time, 4 = all the time). The total treatment adherence scores were computed by summing up the scores for items 1 to 15 after reverse scoring items 2, 3, 6, 8, 10, 14, and 15. The range of possible (or potential) total scores is 15–60. A better TAQ score indicates better treatment adherence. The total scores are categorized into three categories: 15–29 (<50%) indicate a low level of treatment adherence, 30–44 indicate moderate levels of treatment adherence, and 45–60 indicate a high level of treatment adherence (28). The questionnaire was adopted from a previous study by Kritpracha. The TAQ was selected because it comprehensively covers treatment adherence dimensions relevant to dialysis patients, including adherence to hemodialysis, medications, fluid restrictions, and dietary restrictions (29). The TAQ consists of 15 items and demonstrates high stability and internal consistency with a Cronbach's alpha of 0.827, making it an effective outcome measure (30).

### 2.4 Data analysis

The data were cleaned to remove all errors from the data collection. The sample characteristics are presented using descriptive statistics, such as mean, standard deviation, frequency, percentage, range, minimum, and maximum values. The relationship between illness perception and treatment adherence behavior was assessed using linear regression analysis and bivariate Pearson correlation analysis. Differences between groups were analyzed using one-way ANOVA. Data were analyzed using SPSS 21.0 for Windows (SPSS, Inc., Chicago, IL) with statistical significance set at *p* < 0.05.

## 3 Results

### 3.1 Patients’ characteristics

The demographic and health-related characteristics of the 100 patients diagnosed with cardio-renal syndrome are shown in Table 1. The patients had a mean age of 53.46 (SD = 14.24). The majority of the patients were male (63%), older than 40 (73%), unemployed (66%), had no formal education or primary school education (75%), were married (97%), and had insufficient monthly income (99%). The majority of the patients (80%) were diagnosed with cardio-renal syndrome stage 4. Most of the patients (95%) had family members who prepared their diet, and 83% of the patients lived with three to four family members. The clinical characteristics of the study participants are also shown in Table 1. More than half of the patients had been on hemodialysis for less than 5 years (56%), nearly three-quarters had been diagnosed with CRS within the past 5 years (73%), and two-thirds of the patients had hypertension disease (68%).

**Table 1:** Demographic and clinical characteristics (*N* = 100).

Characteristics variable	Frequency (*N*)	Percentage (%)	Mean ± SD
Sex			
Male	63	63	
Female	37	37	
Age			53.46 ± 14.24
40 or older	73	73	
Less than 40	27	27	
Monthly income			
Enough	1	1	
Not enough	99	99	
Marital status			
Not married	3	3	
Married	97	97	
Type of CRS			
Type 1	4	4	
Type 2	12	12	
Type 3	4	4	
Type 4	80	80	
Type 5	0	0	
Education level			
No formal education	34	34	
Primary school	41	41	
Secondary school	15	15	
Tertiary education or above	10	10	
Employment status			
Full-time	20	20	
Part-time	8	8	
Retired	6	6	
Unemployed	66	66	
Number of family members			
Two persons	17	17	
More than two	83	83	
Diet prepared by			
Patient	3	3	
Family member	95	95	
Other	2	2	
Length of diagnosis with CRS			
Less than 5 years	73	73	
5–10 years	18	18	
More than 10 years	9	9	
Length of hemodialysis			
Less than 5 years	56	56	
5–10 years	25	25	
More than 10 years	19	19	
Comorbidities			
Diabetes mellitus	23	23	
Hypertension	68	68	
Other	9	9	

### 3.2 Treatment adherence level

Table 2 summarizes the findings for overall treatment adherence and each dimension. The mean score of overall treatment adherence in the total sample was 2.73 ± 0.22, and most patients (87%) had a poor or low level of treatment adherence. Adherence to hemodialysis and fluid restriction was found to be at a moderate level, while adherence to medication and diet restriction was poor or at a low level (Table 2).

**Table 2:** Status of treatment adherence in four dimensions (*N* = 100).

Treatment adherence dimensions	Low adherence *n* (%)	Moderate adherence *n* (%)	High adherence *n* (%)
Adherence to hemodialysis	2	90	8
Adherence to medication	85	5	10
Adherence to fluid restriction	22	67	11
Adherence to diet restriction	87	12	1
Total	87	12	1

Mean ± SD = 2.73 ± 0.22.

Note: Adherence interpretation: High = 45–60, Moderate = 30–44, Low = 15–29.

### 3.3 Illness perception level

The scores of the eight dimensions of illness perception are presented in Table 3. The total illness perception mean score among the 100 cardio-renal syndrome patients was 6.11 ± 0.78, and 90% of patients perceived CRS as a moderate-to-high threat. The highest mean scores were for the timeline, consequences, identity, illness coherence, and emotion dimensions. According to the findings, patients with CRS perceived high levels of threat in several illness perception dimensions, including consequences (the perceived impact of CRS on their life), timeline (the perceived duration of CRS), coherence (the perceived understanding of CRS), and emotion (the perceived impact of CRS on their emotional status).

**Table 3:** Status of illness threat perception (ITP) in eight dimensions (*N* = 100).

Items	Low ITP *n* (%)	Moderate ITP *n* (%)	High ITP *n* (%)
Consequences	26	7	67
Timeline	9	1	90
Personal control	28	25	47
Treatment control	64	20	16
Identity	3	27	70
Concern	41	14	45
Illness coherence	10	20	70
Emotions	24	22	54
Total	10	57	33

Mean ± SD = 6.11 ± 0.78.

### 3.4 Correlation analysis of illness perception, sociodemographic, clinical characteristics, and treatment adherence

The study’s results revealed no significant correlation between overall illness perception and overall treatment adherence (*r* = 0.003, *p* = 0.975) (Table 4). However, the perceived consequences and perceived timeline subscales of illness perception had a negative correlation with overall treatment adherence (*r* = −0.210, *p* < 0.05; *r* = −0.276, *p* < 0.01, respectively), as shown in Table 5, indicating that a higher level of perceived illness threat results in decreased treatment adherence.

**Table 4:** Correlation analysis of illness perception and treatment adherence among cardio-renal syndrome patients (*N* = 100).

Item	Treatment adherence	
Illness perception	Pearson correlation	0.003
	*p*-value	0.975
	*N*	100

**Table 5:** Pearson correlation between illness perception and treatment adherence in each variable (*N* = 100).

Dimensions of illness perception	Treatment adherence				
	Hemodialysis	Medication	Fluid	Diet	Overall treatment adherence
Consequences	0.049	−0.178	−0.182	−0.037	−0.210^b^
Timeline	0.036	−0.166	−0.274^a^	−0.088	−0.276^a^
Personal control	−0.027	0.092	0.151	0.105	0.186
Treatment control	−0.041	−0.091	−0.004	−0.129	−0.146
Identity	−0.139	−0.040	0.130	0.049	0.036
Concern	−0.005	−0.053	−0.106	0.025	−0.068
Illness coherence	−0.033	0.016	0.158	0.032	0.095
Emotions	−0.243^b^	0.017	0.092	0.000	0.008

^a^ Correlation is significant at the 0.01 level (two-tailed).

^b^ Correlation is significant at the 0.05 level (two-tailed).

As shown in Table 6, the study’s results revealed that income level was positively correlated with adherence, as evidenced by the fact that income was a significant predictor of total adherence (*β* = 11.132, *p* < .001). Furthermore, there was a significant positive correlation between cardiac disorders and treatment adherence (*β* = 4.292, *p* = 0.009), suggesting that patients with cardiac diseases typically have greater adherence rates.

**Table 6:** Linear regression analysis between sociodemographic and clinical characteristics and total adherence (*N* = 100).

Variable	Treatment adherence		
	*t*	95% CI	*p*-value
Age (≥40)	−0.333	−1.628 to 1.162	0.740
Sex (male)	0.330	−1.014 to 1.415	0.743
Marital status	0.737	−1.049 to 2.280	0.464
Education level			
No formal education	−0.002	−1.317 to 1.315	0.998
Secondary school	−0.592	−2.187 to 1.185	0.555
Tertiary education or above	−0.829	−2.800 to 1.155	0.410
Monthly income	4.093	5.712 to 16.552	0.000
Length of diagnosis with CRS (5–10 years)	1.272	−0.427 to 1.933	0.208
Length of hemodialysis (5–10 years)	−0.594	−1.601 to 0.866	0.554
Comorbidity (cardiac comorbidities^a^)	2.667	1.084 to 7.499	0.009

Dependent variable: total treatment adherence; *t* is independent *t*-test, 95% confidence level.

^a^ The presence of cardiac diseases prior to CRS development. *p* ≤ 0.05.

## 4 Discussion

This was the first study in Yemen to assess the levels of illness perceptions and treatment adherence among patients with CRS undergoing dialysis treatment. In addition, no studies have examined the relationship between illness perception and treatment adherence in patients with CRS.

### 4.1 Demographic data and health information

Most of the participants with cardio-renal syndrome were male and older than 40. Al-hwiesh et al. supported these findings by reporting that the median age of individuals with cardio-renal syndrome was 56 (45–68) years, and the majority of them were male in Saudi Arabia (31). Regarding marital status and level of education, most of the participants were married and had either completed elementary school or had no formal education. These findings were in line with a prior study conducted in Yemen (32). Concerning comorbid disease, the majority of the patients had hypertension and diabetes mellitus. These findings were consistent with a previous study by Al-Jarallah (33).

### 4.2 Level of treatment adherence

Overall, treatment adherence was low among cardio-renal syndrome patients, mainly in two dimensions: medication adherence and diet restriction adherence. This may be because they do not know the non-adherence risk or understand the dietary instructions. Furthermore, these treatments are highly demanding and may necessitate a lifetime of therapy sessions, dietary and fluid restriction modifications, polymedication protocols, and follow-up consultations with a healthcare provider. This result was in line with some previous studies (34, 35) that stated that the recommendations for fluid and dietary restrictions were the most challenging for dialysis patients’ adherence to these restrictions. The patient's capability to follow several clinical guidelines and lifestyle modifications is crucial for the success of dialysis in ESRD (36). Patients at risk for or already suffering from cardiovascular disease should participate in cardiac health practices that prevent disease progression and improve their health status (37). A patient may perceive themselves to be fully adherent, even though they may not be taking their medications because the medications were not allotted due to a shortage of some medications and complaints of medication side effects. These findings were consistent with a previous study that showed that 74.7% of participants had poor medication adherence in patients with heart failure in Tanzania (38). Therefore, better treatment adherence should be highlighted in the clinical secondary prevention in patients with CRS.

The low treatment adherence observed in our study aligns with a WHO report indicating that adherence to chronic treatments can be as low as 50% (39). This finding is consistent with previous international research showing similarly low adherence rates in Yemen (40), Spain (41), and India (42). However, it contrasts with studies from Ethiopia (43), Jordan (44), and Saudi Arabia (45), which reported even lower adherence rates of 29%, 27.7%, and 23%, respectively. The low adherence rate in our study highlights a significant public health issue. Variations in findings across studies may stem from differences in research methodologies, patient populations, economic conditions, and the specific instruments used to assess medication adherence.

Our study presents a smaller sample size, which is related to the fact that patient numbers keep dwindling due to the patients not being able to complete all hemodialysis sessions due to financial problems. Furthermore, the number of patients who come to the hemodialysis centers is very small; therefore, making data collection procedures and assessing hemodialysis adherence quite challenging. Moreover, existing studies in the literature support the use of similar durations in studies assessing adherence and related outcomes in hemodialysis patients, indicating that meaningful insights can be gained even within this timeframe (46, 47). However, we acknowledge this as a limitation of our study and suggest that future research should consider longer follow-up periods to fully explore adherence patterns and their evolution over time. This would provide a more comprehensive understanding of the factors influencing adherence in patients with cardio-renal syndrome undergoing hemodialysis.

### 4.3 Illness perception level

The majority of participants had a moderate-to-high level of perceived illness threat. This may be because most of the participants had primary school or no formal education, so the patients with cardio-renal syndrome perceived a high illness threat due to an inadequate understanding of the disease. Patients did not receive enough information from doctors and nurses regarding cardio-renal syndrome and how to control it in the future. This result was in line with a previous study conducted in Indonesia (25). Therefore, there is a critical need to pay attention to patients’ perceptions of their illnesses and to offer them individualized health education or intervention programs to improve their perceptions (48).

The level of perceived illness threat was high regarding the disease identity dimension. Most patients experience shortness of breath, chest pain, edema, and fatigue when experiencing a disease attack. Furthermore, these symptoms affect their daily lives. Changes in the patients’ lives, such as numerous complications, a rigorous pharmaceutical regimen and nutrition, probable dependence on family or friends, and hospitalization, may account for the elevated risk of adverse outcomes. This result was consistent with the findings of previous research studies, which indicated that a high acute/chronic timeline score and a high consequences score are expected because the disease has a chronic course. The high consequences score may be due to the changes in the patients’ lives (49).

Participants with cardio-renal syndrome reported a high level of perceived threat for the timeline or the illness duration dimensions because they perceived cardio-renal syndrome as a chronic condition requiring long-term therapy. High levels of threat for the identity dimension were reported, indicating the perception of severe symptoms. Cardio-renal syndrome is characterized by dyspnea, chest discomfort, edema, and fatigue. When dialysis is performed and medications are taken as prescribed, severe symptoms disappear. Parallel research (30) found that common symptoms experienced by hemodialysis patients are breathlessness, edema, and fatigue.

Participants with cardio-renal syndrome also reported emotional concern, indicating a moderate-to-high level of anxiety, concern, depression, worry about the future, and reluctance to rely on family. A study by Devcich et al. (50) similarly found that individuals emotionally respond to changes after living with the disease. The level of perceived illness threat was high regarding the comprehension of the illness dimension due to most of the patients having an educational level of primary school or no formal education. Moreover, they felt that God had caused the disease and the belief that God had triggered the illness was common.

### 4.4 The relationship between illness perception and treatment adherence

The study's overall results revealed no correlation between treatment adherence score and illness perception. However, for individual dimensions, our results showed that perceived threat from consequences was negatively correlated with total treatment adherence (*r* = −0.210, *p* < 0.05). Furthermore, a negative correlation was found between overall treatment adherence and the perceived threat of the timeline (*r* = −0.276, *p* < 0.01), indicating a higher perception of a threat of impact on their life and poorer treatment adherence behavior or attitude. Although this study showed no association between illness perception and treatment adherence, illness perception remains an important domain of disease management and should not be overlooked (51). As indicated by the results of this study, there is still room for improvement, as there is scope to increase the illness perception score from moderate to a high or perfect score. This demonstrates the necessity of educating patients on the significance of treatment adherence and promoting a thorough awareness of cardio-renal syndrome symptoms to empower patients to keep their illnesses under control. Similarly, a study conducted in Malaysia (49) showed that type two diabetes mellitus patients with high treatment adherence had significantly higher illness coherence scores but significantly lower emotional representations and consequences scores.

Our findings revealed several significant findings in the multivariate linear regression analysis on adherence to treatment and sociodemographic and clinical characteristics. Income level was positively correlated with adherence, as evidenced by the fact that income was a significant predictor of total adherence. This finding is consistent with a previous study that showed that families with lower incomes had a higher risk of poor overall adherence (52). The positive association between monthly income and treatment adherence among patients can be attributed to several factors. First, individuals with higher incomes often have better access to healthcare services, including medications and regular appointments with healthcare providers. This improved access enables them to adhere more closely to prescribed treatment regimens. Second, higher income levels may reduce financial barriers to obtaining medications, leading to consistent usage. In addition, individuals with higher incomes may have greater health literacy and awareness, allowing them to understand the importance of adhering to their treatment plans. Furthermore, there is a noteworthy positive correlation between adherence and cardiac disorders, suggesting that patients with cardiac diseases typically have greater adherence rates. This might be explained by cardiac disease and renal failure often coexisting due to shared risk factors such as hypertension, diabetes, and vascular disease. Patients with both conditions may be more motivated to adhere to their dialysis treatment as it plays a crucial role in managing their overall health, including cardiac function. In addition, dialysis patients with cardiac comorbidities may receive more comprehensive care and monitoring, leading to increased awareness and diligence in following their treatment plans. Furthermore, the complex interplay between renal and cardiac functions underscores the importance of treatment adherence for optimal outcomes, driving patients with both conditions to prioritize their healthcare. This finding was inconsistent with a previous study that found a significant inverse linear association between cardiovascular medication adherence and cardiovascular events (53). However, because they lacked significant predictive ability, a number of medical and demographic variables—such as city, age, education level, length of diagnosis, and type of dialysis—were omitted from the research. In addition, the distance and transportation variables were not taken into account in this study as Yemen has well-established infrastructure.

Yemenis believe that God has predestined health and illness and that seeking medical treatment may be contrary to God's purpose. This mindset can deter people from immediately seeking medical care, resulting in delayed diagnosis and treatment of ailments. This aligns with a study conducted by Freire de Medeiros et al. (54), which showed that religion was significantly associated with adherence to hemodialysis. Therefore, a possible reason for lower adherence might be that most of the participants in our study were Muslims and they believed that their life is destined by God. Illness perceptions and other beliefs, such as spiritual, religious, and cultural views, can influence an individual's actions and have an adverse or beneficial impact on their health and treatment. Cultural ideas about peacefulness after a negative medical test, satisfaction following a medical consultation, and patients’ perceptions of sickness and a future need for related services all play an important role. Thus, disease perceptions determine how an individual copes with the circumstance (for example, receiving treatment) and their emotional response to illness (55). The situation in Saudi Arabia is no different, as a previous study found that cultural and religious views have a substantial impact on healthcare practices in Saudi Arabia and these beliefs can influence people's attitudes, behaviors, and perceptions about health, illnesses, and medical care (56). A previous study on the complex relationship between culture, health, and disease concluded by emphasizing the enduring influence of culture on perceptions of health and disease throughout history (55). Therefore, healthcare providers can promote illness perception and treatment adherence behaviors if they understand these cultural factors and develop nursing interventions that take cultural influences into account.

## 5 Strengths and limitations

The strength of this study is that, to our knowledge, it is the first study to assess the levels of illness perceptions and treatment adherence in a considerable number of cardio-renal syndrome patients treated with dialysis in Yemen and identify the relationship between illness perception and treatment adherence. Despite its strength, there are some limitations. (1) Dependence on self-reported questionnaires may have led to biased results. (2) There was an inadequate sample size due to decreased patient flow during the COVID-19 pandemic. (3) The patients were only from one study area (Hadhramout Province), which makes it difficult to generalize the results to other parts of Yemen or other countries due to social and cultural aspects being an important part of how people perceive illness. (4) While convenience sampling carries the risk of selection bias, we implemented strategic measures to mitigate this issue. These measures aimed to enhance the representativeness of our sample across various subgroups of the population. In addition, we made a concerted effort to recruit patients during different times of the day and across various days of the week to capture a broader cross-section of individuals with different schedules and dialysis routines. Furthermore, inclusion criteria were clearly defined and consistently applied to all potential participants, ensuring that only those who met the established criteria were considered for the study. These results must be confirmed and expanded upon in large sample sizes and multicenter prospective studies. Another limitation could be that cross-sectional studies assess one point in time. Therefore, it might be difficult to show the cause–effect relationship.

## 6 Implication

In clinical settings, understanding the link between how a person thinks about their illness and how well they follow their treatment plan can help nurses create a program to help people with cardio-renal syndrome and ESRD think more positively about their conditions. Furthermore, public health nurses can target cardio-renal health-related behaviors using the concept of disease perception. They can help patients with ESRD and cardio-renal syndrome quit smoking, control their blood pressure, exercise, manage stress, and take medications. They should encourage patients with cardio-renal syndrome and ESRD with limited personal control or low illness concerns to improve cardio-renal health behavior.

## 7 Conclusion

The current study found that most patients with cardio-renal syndrome had poor treatment adherence and a moderate-to-high perception of illness threat. The study showed no significant correlation between overall treatment adherence and illness perception. However, the perceived consequences of the disease and perceived timeline were inversely correlated with treatment adherence. The study’s results revealed that income level is positively correlated with adherence. Furthermore, there is a noteworthy positive correlation between adherence and cardiac disorders. Our results suggest that clinicians should be aware of illness perception dimensions specific to cardio-renal syndrome disease when developing interdisciplinary interventions to assist patients with adjusting to and managing aspects of their condition.

## Data Availability

The original contributions presented in the study are included in the article/Supplementary Material, further inquiries can be directed to the corresponding author.
