# Supplementary material for: The association of illness perception and related factors with treatment adherence among chronic hemodialysis patients with cardio-renal syndrome in Yemen
Source: Front Cardiovasc Med. 2025 Apr 30;12:1432648. doi: 10.3389/fcvm.2025.1432648 (PMC12075227; doi:10.3389/fcvm.2025.1432648)
Supplement: Supplementary file 1 [file Datasheet1.pdf]

Scales used

1.The Demographic and Clinical Data Questionnaire (DCDQ)

This questionnaire consists of two main sections. Please answer all the questions in each section.

Section A: Demographic data

(1) Age: _________________

(2) Gender: ( ) Male ( ) Female

(3) Marital status:
( ) single
( ) married
( ) Divorced / Separated
( ) Widowed

(4) Education:
( ) No formal education
( ) Primary school
( ) Secondary school
( ) Tertiary education or above

(5) Employment status:
( ) Full time Job title: ______________________
( ) Part Time Job title: ______________________
( ) Retired
( ) Unemployed
( ) Housewife

(6) Monthly family income:
1- Sufficient ( )
2- Insufficient ( )

(7) No. of family members living together (excluding patient): _________________

(8) Daily diet prepared by:
( ) Patient
( ) Family member. Please specify: ___________________
( ) Maid
( ) Other. Please specify: _____________________

Section B: Clinical data

(1) Weight: Kg

(2) Blood Pressure: / mmg

(3) Blood Urea level: mg/dl

(4) Month(s) diagnosed with cardiorenal syndrome disease:_____year(s)_month(s).

(5) Length of receiving hemodialysis or peritoneal dialysis: ____year(s)__month(s).

(6) Types of dialysis: ( ) Hemodialysis ( ) peritoneal dialysis

(7) Co-morbidities:

( ) No ( ) Heart disease

( ) Diabetes mellitus ( ) Hypertention

( ) Hyperparathyroidism ( ) Other. Please specify: _______________

(8) Cardiorenal Syndrome Disease stage;

( ) stage 1

( ) stage 2

( ) stage 3

( ) stage 4

( ) stage 5

2.The Treatment Adherence Questionnaire (TAQ)

This survey asks for your opinion about how well you follow your dialysis treatment schedule and about medical recommendations related to medication, diet, and fluid intake. This information will help us to understand if you have difficulty following your dialysis treatment, medication regimen, fluid restriction, and recommended diet. This questionnaire consists of four main sections. Please answer every question by marking the appropriate box.

No	Statement	Never	Sometimes	Most of the times	All of the times
**Section A: Adherence to Hemodialysis**					
**1**	I attended my dialysis treatment regularly.				
**2**	I have shortened my dialysis time.				
**Section B: Adherence to Medication**					
**3**	I missed the prescribed medications.				
**4**	I took my medications even though I have problem due to side effect of the medications.				
**5**	I took my prescribed medications even though I do not have any symptoms.				
**6**	I stopped taking medication.				
**Section C: Adherence to Fluid Restriction**					
**7**	I followed the fluid restriction recommendation.				
**8**	I took water as much as I want.				
**9**	I managed my thirst, for example by staying in cool place, sipping my beverage, or using the ice cube.				
**10**	I took food with hidden fluids, for example soup or ice creams.				
**Section D: Adherence to Diet Restriction**					
**11**	I followed the diet recommendation.				
**12**	I took high protein foods, for example 2 matchbox size of meats, fish, or 1 drumstick of chicken every day.				
**13**	I avoid foods containing salt.				
**14**	I took high phosphate foods, for example beans, dried vegetables or fruits, or chocolate.				
**15**	I took high potassium foods, for example bananas, papayas or oranges.				

3.The Brief Illness Perception Questionnaire (BIPQ)

No.	For the following questions, please circle the number that best corresponds to your views:
1.	How much does your cardiorenal syndrome disease affect your life?<br>0 1 2 3 4 5 6 7 8 9 10<br>no affect — severely affects my
2.	How long do you think your cardiorenal syndrome disease will continue?<br>0 1 2 3 4 5 6 7 8 9 10<br>a very short — forever
3.	How much control do you feel you have over your disease?<br>0 1 2 3 4 5 6 7 8 9 10<br>absolutey no control — amount of control — extreme
4.	How much do you think your treatment can help your disease?<br>0 1 2 3 4 5 6 7 8 9 10<br>not at all — extremely — helpful
5.	How much do you experience symptoms from your disease?<br>0 1 2 3 4 5 6 7 8 9 10<br>no symptom — many severe — symptoms
6.	How concerned are you about your disease?<br>0 1 2 3 4 5 6 7 8 9 10<br>not at all concerne — extremely concerned
7.	How well do you feel you understand your disese ?<br>0 1 2 3 4 5 6 7 8 9 10<br>don't understand at — very clearly — understand
8.	How much does your disease affect you emotionally? (e.g. does it make you angry, scared, upset or depressed?<br>0 1 2 3 4 5 6 7 8 9 10<br>no affect — affects my life — severely

9.	Please list in rank-order the three most important factors that you believe caused <u>your disease</u>. The most important causes for me:-<br><br>**1.** ---<br>**2.** ---<br>**3.** ---
